# Urinary beta 3-adrenoceptor as a diagnostic biomarker for overactive bladder in women

**DOI:** 10.1038/s41598-023-46786-6

**Published:** 2023-11-08

**Authors:** Ching-Chung Liang, Wu-Chiao Hsieh, Tsia-Shu Lo, Ting-Xuan Huang, Yi-Chun Chou, Jing-Yi Huang, Yung-Hsin Huang

**Affiliations:** 1 Department of Obstetrics and Gynecology, Chang Gung Memorial Hospital at Linkou, 5, Fu-Shin Street, Kweishan, Taoyuan, 333 Taiwan; 2 College of Medicine, Chang Gung University, Taoyuan, Taiwan; 3 Center for Big Data Analytics and Statistics, Chang Gung Memorial Hospital at Linkou, Taoyuan, Taiwan

**Keywords:** Biomarkers, Medical research, Urology

## Abstract

This study was to investigate urinary beta 3-adrenoceptor concentration as a biomarker for overactive bladder (OAB) and predictor of treatment outcomes in women receiving the beta 3-adrenoceptor agonist mirabegron. The study comprised 50 women identified with OAB and 35 women considered as healthy controls. All women with OAB received daily dosage of 50 mg of mirabegron for 12 weeks. Bladder diaries, OAB-related questionnaires, and global response assessment scale (GRAS) data were collected. Urinary beta 3-adrenoceptor concentration was measured through enzyme-linked immunosorbent assay. All OAB-related questionnaires and GRAS indicated improved posttreatment urinary health. After mirabegron treatment, the frequency of micturition and urgency episodes decreased, but the urinary beta 3-adrenoceptor/creatinine (Cr) ratio increased. The urinary beta 3-adrenoceptor/creatinine ratio was identified as a sensitive biomarker for OAB with a confidence interval of 0.656 to 0.856 (*p* < 0.001). A negative correlation (− 0.431, *p* = 0.040) between this biomarker and health-related quality of life (HRQL) scores. The Beta 3-adrenoceptor/Cr levels increased significantly in the treatment-responsive group, while they remained unchanged in the unsatisfactory outcome group. This study shows that 12 weeks of mirabegron treatment improves OAB symptoms and HRQL. Furthermore, urinary beta 3-adrenoceptor concentration may be a diagnostic biomarker for OAB.

## Introduction

Overactive bladder (OAB) is a prevalent condition known to have a detrimental impact on the quality of life (QOL). Its prevalence varies up to 16–34% in both Asian and Western countries, collectively affecting over 400 million individuals^1–4^. The clinical symptoms of OAB include urgency and frequency, with or without urge incontinence^5,6^. However, the underlying pathophysiology of OAB remains incompletely elucidated. Normal bladder emptying occurs after the release of acetylcholine from parasympathetic nerves, which causes the detrusor muscle to contract, the urethral sphincter to relax, and the bladder outlet to open^7^. In the case of OAB, dysfunction in the afferent pathways can result in overactivity in the efferent pathways and thus lead to involuntary detrusor contractions. The activation of the muscarinic receptors might possibly be the source of these contractions^7,8^. Oral antimuscarinic agents represent the primary treatment modality for OAB, exhibiting notable efficacy. Nonetheless, their application is accompanied by elevated incidences of adverse events, including constipation and dry mouth^9^. In contrast to oral antimuscarinic agents, the beta 3-adrenoceptor agonist (such as mirabegron) is associated with fewer side effects while demonstrating comparable efficacy to antimuscarinics^10–12^.

Beta 3-adrenoceptors actively induce detrusor muscle relaxation, facilitating urine storage in the bladder^13–18^. Upon sympathetic nerve stimulation, adrenoceptors located in the urothelium and detrusor (predominantly beta 3 subtype) become activated^13^. This activation leads to the inhibition of afferent bladder activity, detrusor muscle relaxation, and enables urine storage^8,14–16^. In experimental studies, it has been shown that beta 3-adrenoceptor agonists can inhibit the afferent activity of A delta- and C-fiber nerves when the bladder is enlarged^17^. Furthermore, these agonists can reduce bladder contraction but do not have a significant effect on bladder pressure reduction^18^.

A multitude of biomarkers have been proposed to aid in the diagnosis of OAB and to predict the therapeutic effects to OAB treatment^19,20^. Among these biomarkers, urinary nerve growth factor has proven to be helpful in assessing the effect of antimuscarinic therapy on OAB^21–24^. Although mirabegron is progressively utilized by physicians as a primary treatment for OAB, no biomarkers have been identified thus far to predict the therapeutic outcomes of beta 3-adrenoceptor agonist therapy in OAB. In an animal study, we demonstrated that the immunoreactivity and mRNA levels of beta 3-adrenoceptor were increased in the bladder of rats with spinal cord injury. Bladder dysfunction in spinal cord-injured rats can be improved by amniotic fluid stem cell transplantation^25^. We speculated that urinary beta 3-adrenoceptor concentration is linked to OAB and could serve as a diagnostic marker for this condition with the possibility of its potential use as a predictive indicator for treatment outcomes in OAB therapy. Thus, the primary objective of our study was to assess the potential of urinary beta 3-adrenoceptor concentration as a biomarker for diagnosing OAB and predicting treatment outcomes following mirabegron treatment.

## Methods

### Participants

From May 2021 to June 2022, female patients with OAB who attended the incontinence clinic at our tertiary hospital were invited to participate in a prospective and nonrandomized controlled trial. All invited patients were asked to complete a 3-day bladder diary. Only women experiencing more than eight episodes of micturition and at least one episode of urgency or urge incontinence per day were diagnosed with OAB^5^. OAB symptoms must persist for more than 3 months. All eligible subjects received oral mirabegron 50 mg once daily for 12 weeks. Exclusion criteria encompassed patients who had taken any anti-OAB medication within the preceding three months, those with uncontrolled hypertension, pelvic organ prolapse, stress urinary incontinence, interstitial cystitis, severe constipation, a history of unsuccessful prior OAB drug therapy, those who had undergone hysterectomy and pelvic reconstructive surgery for stress urinary incontinence or pelvic organ prolapse, or those who were pregnant. Women who came to our hospital for Pap smear and without urinary tract infection, chronic urological diseases or OAB symptoms were invited to serve as controls. All participants provided informed consent. This study was approved by the institutional review board approved of our hospital (Approval Number: 202001412B0), and registered at ClinicalTrials.gov (Identifier: NCT04693897).

### Study design

Pretreatment evaluations included recording the participants’ general medical and obstetric histories and collecting urine cultures and 3-day bladder diaries. Urinalysis, pelvic examination, and urodynamic testing were conducted. Changes in lower urinary tract symptoms and health-related QOL (HRQL) from baseline to 12 weeks after mirabegron treatment were assessed by a research nurse from our outpatient department on the basis of three questionnaires: the Overactive Bladder Symptom Score (OABSS) questionnaire^26^; Overactive Bladder Questionnaire Short Form (OAB-qSF), which comprises a 6-item symptom bother scale and a 13-item HRQL scale^27^; and the 12-Item Short Form Survey (SF-12)^28^. After 12 weeks of mirabegron treatment, the patients rated the changes in the severity of their bladder symptoms by using the global response assessment scale (GRAS); scale 1–5 indicates significantly worse (no improvement), somewhat worse (0–25% improvement), no change (25–50% improvement), somewhat improved (50–75% improvement), and significantly improved (75–100% improvement), respectively^29^. A response of somewhat improved or significantly improved was considered to indicate treatment success.

### Measurement of urinary beta-3 adrenoceptor concentration

Urine samples were collected when controls and patients with OAB experienced a comfortable desire to void. Urinary beta 3-adrenoceptor concentrations were measured in all participants before treatment and in patients of OAB after 12 weeks of mirabegron treatment. Urine samples from all participants were iced immediately after collection until being centrifuged at 3000 rpm for 10 min at 4 °C. The supernatant was divided into aliquots in 1.5-mL tubes and preserved in a − 80 °C freezer. Concurrently, 3 mL of urine was used to measure the urinary creatinine (Cr). Urinary beta 3-adrenoceptor concentration was determined with an enzyme-linked immunosorbent assay (ELISA) kit (MyBioSource, San Diego, CA, USA). The amount of beta-3 adrenoceptor in each urine sample was determined from a beta 3-adrenoceptor standard curve. All samples were tested in triplicate and the values were averaged. Urinary beta 3-adrenoceptor concentration was normalized to urinary Cr concentration, and this ratio (beta 3-adrenoceptor/Cr) was used for analysis.

### Statistical analysis

Clinical data are presented as mean ± standard deviation or percentage, depending on the characteristics of the variables. For parametric data, statistical comparisons were conducted using either Student’s t-test or one-way analysis of variance (ANOVA), while nonparametric data were analyzed with either the Mann–Whitney U test or the Kruskal–Wallis test. Categorical data were analyzed with either the chi-square test or the Fisher exact test. The questionnaires scores and bladder diary parameters of normal controls, patients with and without 12-week mirabegron treatment were compared through the Kruskal–Wallis test followed by Dunn's post hoc test. The paired t test and Wilcoxon signed-rank test were used to compare pretreatment and posttreatment values. Spearman correlation analyses were conducted to analyze the relationships among characteristics (age, BMI and parity), questionnaires scores, urinary symptoms (daily frequency of micturition and urgency or urge incontinence), uroflowmetry parameters, and urinary beta 3-adrenoceptor/Cr ratio. OAB diagnosis based on urinary beta 3-adrenoceptor was analyzed using the receiver operating characteristic (ROC) curve. All statistical analyses were conducted with SPSS version 22.0 (IBM Co., Armonk, NY, USA). A *p* value of < 0.05 was considered statistically significant.

## Results

### Characteristics of patients

Fifty patients with OAB and 35 controls were included in the study. Twenty-three patients completed 12 weeks of mirabegron treatment, and 27 patients did not receive treatment because they dropped out after the first visit. Table 1 presents the characteristics of non-treated and treated patients and controls which shows the patient characteristics did not differ significantly among the three groups.

**Table 1:** Characteristics of normal controls, patients with and without 12-week mirabegron treatment.

	Normal (n = 35)	Non-treated OAB (n = 27)	Treated OAB (n = 23)	*p* value^a^
Age (y)^b^	50.4 ± 11.2	52.5 ± 11.3	57.7 ± 12.5	0.068
BMI (kg/m^2^)^b^	22.7 ± 2.7	23.8 ± 3.5	23.0 ± 2.4	0.341
Married	24 (68.6%)	20 (74.1%)	15 (65.2%)	0.787
Parity^c^	1.7 ± 0.9	2.0 ± 1.5	2.2 ± 1.4	0.379
NSD	19 (54.3%)	19 (70.4%)	15 (65.2%)	0.409
Menopause	19 (54.3%)	18 (66.7%)	15 (65.2%)	0.549
Prior HT	2 (5.7%)	5 (18.5%)	3 (13.0%)	0.300
Smoking	2 (5.7%)	1 (3.7%)	2 (8.7%)	0.854
Medical diseases	7 (20.0%)	2 (7.4%)	5 (21.7%)	0.301
Prior Gyn surgeries	22 (62.9%)	17 (63.0%)	15 (65.2%)	0.981

Data are presented as mean ± standard deviation and n (%).

OAB, Overactive bladder; BMI, Body mass index; NSD, Normal vaginal delivery; HT, Hormonal therapy; Gyn, Gynecological.

Medical diseases include hypertension, diabetes and heart diseases.

Prior Gyn surgeries include uterine myomectomy, adnexal mass resection, fallopian tube ligation and cesarean section.

^a^Chi-squared test and the Fisher's exact test.

^b^One-way analysis of variance (ANOVA).

^c^Kruskal–Wallis test.

*p < 0.05.

### Mirabegron improves OAB questionnaire scores and urinary symptoms

Table 2 summarizes the baseline OAB questionnaire scores in the OAB and control groups as well as the changes in scores after 12 weeks of mirabegron treatment in the OAB group. The mean total OABSS and OAB-qSF bother and HRQL scores in the OAB group were higher than those in the control group (*p* < 0.001), but there were no significant differences in SF-12 scores and GRAS.

**Table 2:** The questionnaires scores, urinary symptoms and uroflowmetry parameters in normal controls, non-treated and treated OAB groups before mirabegron treatment.

	Normal (n = 35)	Non-treated OAB (n = 27)	Treated OAB (n = 23)	*p* value^a^
OABSS	4.83 ± 0.62	11.00 ± 2.62	12.00 ± 2.97	< 0.001*^#†^
OAB-qSF symptom bother	6.57 ± 0.95	19.63 ± 8.24	24.52 ± 6.08	< 0.001*^#†^
OAB-qSF HRQL	14.06 ± 2.35	44.07 ± 12.18	44.48 ± 9.36	< 0.001*^#†^
SF-12	32.09 ± 1.27	32.19 ± 2.04	32.91 ± 2.04	0.108
GRAS	3.00 ± 0.00	3.00 ± 0.00	3.00 ± 0.00	1.000
Micturitions/24 h	7.34 ± 1.14	15.23 ± 6.73	15.65 ± 5.50	< 0.001*^#†^
Urgency episodes/24 h	0.00 ± 0.00	1.67 ± 0.97	1.79 ± 0.84	< 0.001*^#†^
Uroflowmetry				
Qmax^b^ (mL/s)	–	15.65 ± 6.99	13.67 ± 5.32	0.271
Capacity^c^ (mL)	–	285.65 ± 147.15	313.87 ± 145.13	0.471
RU^c^ (mL)	–	51.56 ± 74.39	56.17 ± 66.84	0.440

Data are presented as mean ± standard deviation.

OAB, Overactive bladder; OABSS, Overactive bladder symptom score; OAB-Qsf, Overactive bladder questionnaire short form; HRQL, Health-related quality of life scales; SF-12, Short Form Survey-12; GRAS, Global Response Assessment Scale; Qmax, Maximum flow rate; RU, Residual urine.

^a^Kruskal–Wallis test with Dunn's test.

^b^Student t-test.

^c^Mann–Whitney U-test.

*p < 0.05.

^#^*p* < 0.05 Normal vs Non-treated OAB.

^†^*p* < 0.05 Normal vs Treated OAB.

Table 3 shows that after 12 weeks of mirabegron treatment, the mean total OABSS and OAB-qSF symptom bother and HRQL scores significantly decreased (*p* < 0.001) in 23 treated patients, whereas mean SF-12 scores did not change significantly. Additionally, GRAS scores significantly improved after treatment (*p* < 0.001). The mean numbers of micturition and urgency episodes per day in the OAB group were significantly lower after treatment (*p* < 0.001). After mirabegron treatment, 17 patients were successfully treated and 6 patients had unsatisfactory treatment results. The success rate of OAB treatment is 73.9%.

**Table 3:** Changes in questionnaires scores and urinary symptoms before and after 12 weeks of mirabegron treatment in patients with OAB.

	OAB before treatment	OAB after treatment	*p* value^a^
	(n = 23)	(n = 23)	
OABSS^b^	12.00 ± 2.97	7.52 ± 2.04	< 0.001*
OAB-qSF symptom bother	24.52 ± 6.08	13.39 ± 6.39	< 0.001*
OAB-qSF HRQL	44.48 ± 9.36	26.48 ± 12.35	< 0.001*
SF-12	32.91 ± 2.04	32.83 ± 1.53	0.775
GRAS	3.00 ± 0.00	4.43 ± 0.95	< 0.001*
Micturitions/24 h	15.65 ± 5.50	10.30 ± 3.00	< 0.001*
Urgency episodes/24 h	1.79 ± 0.84	0.45 ± 0.39	< 0.001*

Data are presented as mean ± standard deviation.

OABSS, Overactive bladder symptom score; OAB-qSF, Overactive bladder questionnaire short form; HRQL, Health-related quality of life scales; SF-12, Short Form Survey-12; GRAS, Global response assessment scale.

^a^Wilcoxon signed-rank test.

^b^Paired t-test.

*p < 0.05.

### Urinary beta 3-adrenoceptor levels distinguish OAB from controls

In Table 4, the mean baseline urinary beta 3-adrenoceptor/Cr ratio showed a significant reduction in the total OAB group or treated OAB group compared with the normal control group (*p* < 0.001 and *p* = 0.011, respectively). No significant difference was observed in baseline urinary beta 3-adrenoceptor/Cr ratio between the hypersensitive bladder group (n = 39) and detrusor overactivity group (n = 11). Among the 23 treated patients, no statistical differences were found in baseline urinary beta 3-adrenoceptor/Cr ratio between the hypersensitive bladder group (n = 18) and detrusor overactivity group (n = 5).

**Table 4:** Urinary beta 3-adrenoceptor/Cr levels in normal controls, OAB Baseline and subgroups.

	Urinary beta-3 adrenoceptor/Cr (pg/mg)	*p* value^a^
Overall^b^		
Normal (n = 35)	1.98 ± 0.90	
Total OAB baseline (n = 50)^c^	1.25 ± 0.70	< 0.001*
Treated OAB baseline (n = 23)^d^	1.25 ± 0.76	0.011*
OAB Baseline		0.748
Non-treated OAB (n = 27)	1.25 ± 0.66	
Treated OAB (n = 23)	1.25 ± 0.76	
Types of Subgroup—OAB Baseline (n = 50)		0.861
Detrusor overactivity (n = 11)	1.22 ± 0.73	
Hypersensitive bladder (n = 39)	1.26 ± 0.71	
Types of Subgroup—Treated OAB (n = 23)		0.456
Detrusor overactivity (n = 5)	1.41 ± 0.76	
Hypersensitive bladder (n = 18)	1.20 ± 0.78	
Treated OAB		< 0.001*
OAB before treatment (n = 23)	1.25 ± 0.76	
OAB after treatment (n = 23)	4.41 ± 3.72	
Before treatment		0.401
Successful treatment (n = 17)	1.20 ± 0.84	
Unsatisfactory result (n = 6)	1.40 ± 0.54	
After treatment		0.010*
Successful treatment (n = 17)	5.35 ± 3.88	
Unsatisfactory result (n = 6)	1.73 ± 1.04	

Data are presented as mean ± standard deviation.

^a^Mann–Whitney U-test.

^b^Kruskal–Wallis test.

^c^Mann–Whitney U-test, Normal vs Total OAB baseline.

^d^Mann–Whitney U-test, Normal vs Treated OAB baseline.

*p < 0.05.

**Figure 1:**
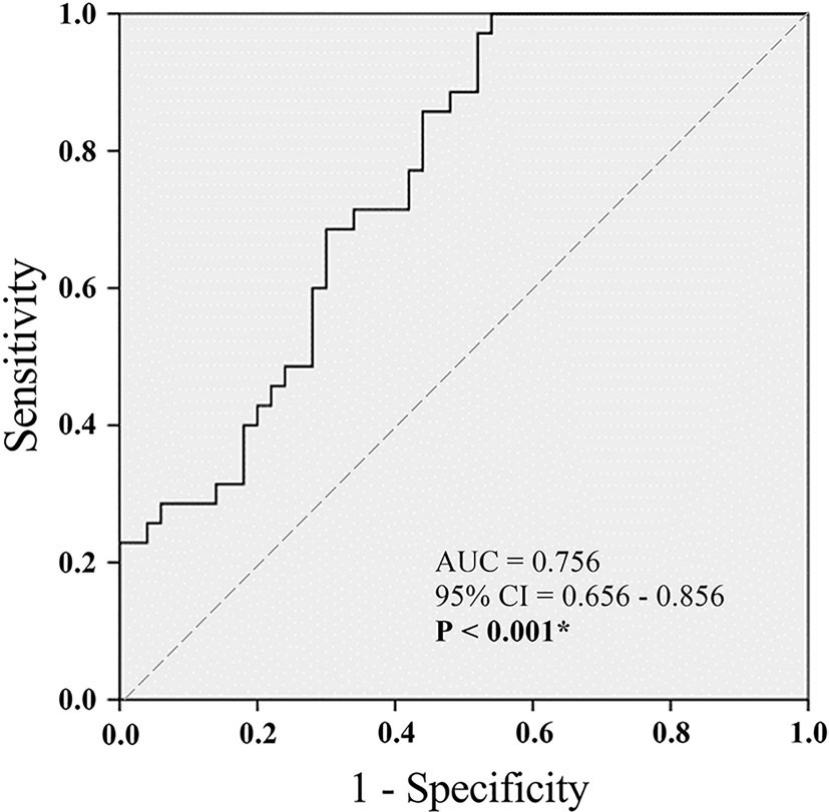
Receiver-operator characteristics (ROC) curve for identifying OAB on the basis of urinary beta 3-adrenoceptor/Cr ratio. A larger area under the curve for the urinary beta 3-adrenoceptor/Cr level of the normal control group was calculated compared to the OAB group. AUC, area under the curve; CI, confidence interval.

The area under ROC curve (AUC) for predictive potential of urinary beta 3-adrenoceptor/Cr ratio was assessed. The AUC for the control group (AUC = 0.756, 95% confidence interval = 0.656–0.856; *p* < 0.001) was significantly greater than that of OAB group (Fig. 1). The optimal cutoff based on the Youden index for the urinary beta 3-adrenoceptor/Cr ratio^30^ was 0.995. Using this cutoff provided sensitivity of 100.0% and specificity of 46.0%. The positive predictive value was 59.7%, while the negative predictive value achieved 100%.

### Urinary beta 3-adrenoceptor levels for predicting treatment outcome

**Figure 2:**
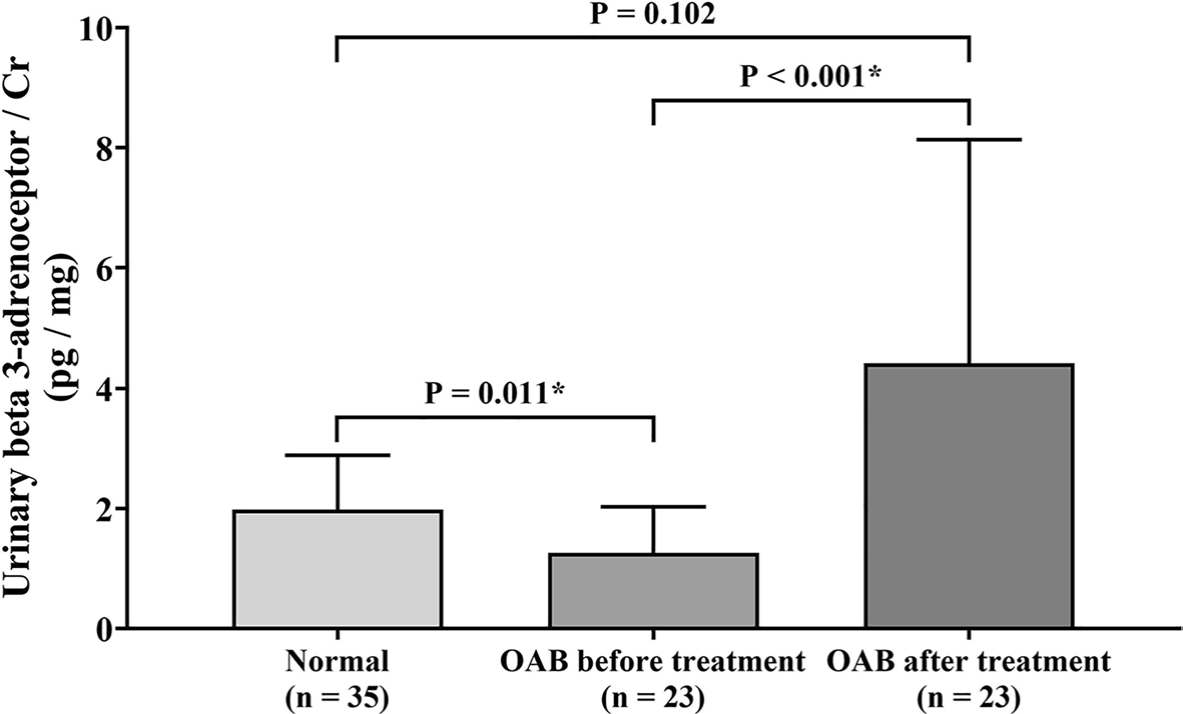
Histogram of urinary beta 3-adrenoceptor levels in normal controls, OAB before and after mirabegron treatment. The mean urinary beta 3-adrenoceptor/Cr level in the OAB group was lower than that in the normal control group, and it was significantly increased after mirabegron treatment. Cr, creatinine.

In the treated OAB group, after 12 weeks of mirabegron treatment, the mean urinary beta 3-adrenoceptor/Cr ratio was significantly increased compared with before treatment (*p* < 0.001) (Fig. 2 and Table 4). Comparing baseline urinary beta 3-adrenoceptor/Cr ratio between the successful treatment group (n = 17) and unsatisfactory result group (n = 6), no statistically difference was observed between both the groups. However, after 12 weeks of mirabegron treatment, beta 3-adrenoceptor/Cr levels were significantly increased in the successful treatment group but not in unsatisfactory result group (*p* < 0.001) (Table 4).

### OAB-qSF HRQL scores correlate with urinary beta 3-adrenoceptor levels

Spearman correlation analyses demonstrated that OAB-qSF HRQL score was negatively correlated with urinary beta 3-adrenoceptor/Cr level (correlation coefficient = − 0.431, *p* = 0.040) in the patients with OAB after mirabegron treatment (Table 5).

**Table 5:** Correlation coefficients between characteristics, questionnaires scores and urinary symptoms, uroflowmetry parameters, and urinary beta 3-adrenoceptor/Cr levels in OAB patients with and without 12-week mirabegron treatment.

	Urinary beta 3-adrenoceptor/Cr (pg/mg)		
	Non-treated OAB before treatment (n = 27)	Treated OAB before treatment (n = 23)	Treated OAB after treatment (n = 23)
	r_s_	r_s_	r_s_
Age (y)	0.174	0.292	0.407
BMI (kg/m^2^)	− 0.170	0.424*	− 0.171
Parity	− 0.047	0.361	0.391
OABSS	− 0.055	0.143	− 0.299
OAB-qSF symptom bother	− 0.003	0.211	− 0.347
OAB-qSF HRQL	0.136	0.403	− 0.431*
SF-12	0.136	− 0.044	− 0.312
GRAS	0.000	0.000	0.367
Micturitions/24 h	− 0.151	0.091	− 0.112
Urgency episodes/24 h	− 0.133	0.162	− 0.385
Uroflowmetry			
Qmax (mL/s)	0.007	− 0.177	0.244
Capacity (mL)	0.161	0.052	− 0.190
RU (mL)	0.114	0.310	− 0.210

OAB, Overactive bladder; BMI, Body mass index; OABSS, Overactive Bladder Symptom Score; OAB-qSF, Overactive Bladder Questionnaire Short Form; HRQL, health-related quality of life scales; SF-12, Short Form Survey-12; GRAS, Global Response Assessment Scale; Qmax, maximum flow rate; RU, residual urine.

r_s_: Spearman's correlation coefficient.

*p < 0.05.

## Discussion

We discovered that the mean urinary beta 3-adrenoceptor/Cr ratio in OAB patients was lower when compared to control group. A urinary beta 3-adrenoceptor/Cr cutoff of 0.995 resulted in the positive predictive value of 59.7% with the negative predictive value of 100% for OAB diagnosis. Our data indicated a significant increase in urinary beta 3-adrenoceptor level after mirabegron treatment, but the cellular mechanisms underlying this increase have not been elucidated. Prior research has indicated a substantial presence of beta 3-adrenoreceptors within the bladder, notably in detrusor muscle and urothelium^13,31,32^. In the storage phase of micturition, norepinephrine released from sympathetic nerves, engages with bladder beta 3-adrenoceptors, inducing relaxation of the detrusor muscle^33^. We propose a hypothesized mechanism here: Physiologically, during storage phase of micturition, bladder distension can stretch the urothelium and detrusor muscle, and the release of urinary beta 3-adrenoceptor can activate bladder relaxation and increase bladder volume. However, in OAB bladders, beta 3-adrenoceptor production may be limited by pathological detrusor contraction during storage phase, resulting in lower urinary beta 3-adrenoceptor levels compared with normal controls. Previous studies have revealed that in OAB-affected bladders, beta 3-adrenoreceptor agonists preferentially inhibit pathologically increased detrusor tone during bladder filling, rather than physiological detrusor contraction during micturition, which may result in increased urinary beta 3-adrenoceptor levels^34,35^. In vitro studies using human bladder strips have shown that the activation of beta 3-adrenoceptor induces bladder relaxation through the adenylyl cyclase pathway and subsequent formation of cyclic adenosine monophosphate. This may indicate basis of therapeutic effect of beta 3-adrenoceptor agonists in OAB^21,22,32^. In clinical practice, beta 3-adrenoceptor agonists boost bladder capacity without concomitant varies in voiding detrusor pressure that enhance postvoid residual volume or decrease detrusor contractility^36^.

Our results showed that urinary beta 3-adrenoceptor/Cr levels were significantly increased in OAB patients after 12 weeks of mirabegron treatment compared with before treatment. In further analysis, we found that there was no statistical difference in the baseline urinary beta 3-adrenoceptor/Cr ratio between the successful treatment group and unsatisfactory result group. However, after mirabegron treatment, the urinary beta 3-adrenoceptor/Cr levels in 17 patients with successful treatment were higher than those in 6 patients with unsatisfactory treatment results. Among 23 treated patients, OAB-qSF HRQL scores were negatively correlated with urinary beta 3-adrenoceptor/Cr levels, meaning that patients with improved HRQL after mirabegron treatment had higher beta 3-adrenoceptor/Cr ratio. Other than OAB-qSF HRQL score, no correlation was observed between the changes of urinary beta 3-adrenoceptor/Cr ratio and the changes of clinical data after mirabegron treatment. To summarize the results of this study on beta 3-adrenoceptor, the urinary beta 3-adrenoceptor/Cr ratio is not a valid predictive tool for the outcomes of beta 3-adrenoceptor agonist therapy. However, this ratio was correlated with OAB-qSF HRQL scores and could predict improvements in QOL after mirabegron treatment.

The results demonstrate that mirabegron is effective for OAB treatment. Twenty-three patients with OAB were treated with mirabegron for 12 weeks, with a success rate of 73.9%. Daily 50-mg doses of mirabegron are as effective as antimuscarinic therapy and having only few side effects^10,37^. Multiple clinical trials have demonstrated the effectiveness and safety of mirabegron in the treatment of OAB^38–40^. A pooled analysis of three phase III placebo-controlled studies indicated that daily dose of 50 mg mirabegron was associated with a favorable safety profile and significant reductions in OAB symptoms, including the frequency of incontinence episodes and frequency and urgency of urination^38^. A recent study of mirabegron in the adults with OAB indicated the medication’s safety and efficacy for different age groups and sexes^41^. The same study demonstrated greater improvements from baseline with mirabegron than with the placebo in mean daily number of episodes micturition, incontinence, urgency, and nocturia and voided volume^41^. In this study, the mean numbers of daily micturition and urgency episodes were significantly lower after 12 weeks of mirabegron treatment. However, some patients might not benefit from mirabegron monotherapy and need combination of mirabegron with antimuscarinics^42,43^. Some investigators suggest that in patients with OAB refractory to medical therapy, videourodynamic studies should be used to evaluate the presence of bladder outlet obstruction to guide effective treatment and prevent unnecessary surgical intervention^44^.

In the present study, OAB-qSF symptom bother and HRQL scores were surpassing in OAB group (*p* < 0.001) than normal control group, but SF-12 scores did not differ significantly. Because of their distinct assessment methods, previous studies have shown inconsistent results on the effectiveness of mirabegron for improving the HRQL of patients with OAB^12,39^. Kuo et al. reported that 50 mg of mirabegron had no significant effect on HRQL as determined from the King’s Health Questionnaire, which analyzes the effect of urinary incontinence on QOL^12^. However, significant enhancement was observed in HRQL outcomes based on OAB questionnaires and patients’ perceptions of their bladder condition^39,40^. In addition, visual analog scale results in previous studies significantly indicated improvements after mirabegron treatment^39,40^. The present study assessed patient-reported symptom response and found that GRAS scores improved significantly after mirabegron treatment.

To the extent of our current knowledge, this is the first clinical trial delving into the influence of mirabegron treatment on urinary beta 3-adrenoceptor concentration among women afflicted with OAB. There are various restrictions with this study that must be taken into account when interpreting the results. Firstly, the number of cases and follow-up period were limited by the COVID-19 pandemic. Second, no placebo group was employed in the design. Third, only women were recruited. Men and women may exhibit differences in their OAB symptoms, symptom sensitivity, and HRQL as well as the relationships of these factors with urinary biomarkers^45^. Fourth, several factors may alter urinary beta 3-adrenoceptor levels, including different bladder volumes, different urgency severity, urine sample storage, and the ELISA methodology for measuring beta 3-adrenoceptor. In this study, to reduce the influence of different urgency severity on urinary beta 3-adrenoceptor levels, urine samples were collected when participants experienced a comfortable desire to void. Additionally, beta 3-adrenoceptor was stored at 4 °C to prevent protein degradation, instability, and microbial growth. In order to establish urinary beta 3-adrenoceptor levels as a potential biomarker for OAB, additional research is required. This will entail the standardization of urine sample collection, the recruitment of a larger number of patients, and a longer follow-up period to validate our observations.

## Conclusion

Patients with OAB exhibit a significantly reduced urinary beta 3-adrenoceptor/Cr ratio, which subsequently experiences a marked increase following mirabegron treatment. In addition, this ratio is an adequate biomarker for OAB diagnosis but not a valid tool predictor of the outcomes of beta 3-adrenoceptor agonist therapy.

## Data Availability

Authors, without undue reservation. Professor Ching-Chung Liang should be contacted at ccjoliang@cgmh.org.tw if anyone wants to request the data from this study.
